# Population-level interventions targeting risk factors of diabetes and hypertension in South Africa: a document review

**DOI:** 10.1186/s12889-021-11910-6

**Published:** 2021-12-14

**Authors:** Jeannine Uwimana - Nicol, Lynn Hendricks, Taryn Young

**Affiliations:** 1 Centre for Evidence based Healthcare, Division of Epidemiology and Biostatistics, Department of Global Health, Faculty of Medicine and Health Sciences, Stellenbosch University, Cape Town, South Africa; 2 College of Medicine and Health Sciences, School of Public Health, University of Rwanda, Kigali, Rwanda; 3 Social, Methodological, Innovative, Kreative, Centre for Sociological Research, Faculty of Social Sciences, Katholieke Universiteit Leuven, Leuven, Belgium

**Keywords:** Population-based interventions, Situation analysis, Diabetes, Hypertension, South Africa, Document review

## Abstract

**Background:**

South Africa bears an increasing burden of non-communicable diseases (NCDs), particularly diabetes, cardiovascular diseases, and cancer. The objective of this study was to identify which population-level interventions, implemented at the level of governmental or political jurisdictions only, targeting risk factors of diabetes and hypertension were included in policies in South Africa. We also looked at whether these have been implemented or not.

**Methods:**

A review of relevant reports, journal articles, and policy documents was conducted. Documentation from government reports that contains information regarding the planning, implementation and evaluation of population-level interventions targeting diabetes and hypertension were considered, and various databases were searched. The identified population-level interventions were categorized as supportive policies, supportive programs and enabling environments according to the major risk factors of NCDs i.e., tobacco use, harmful consumption of alcohol, unhealthy diet/nutrition and physical inactivity, in accordance with the WHO ‘Best buys’. A content document analysis was conducted.

**Results:**

The source documents reviewed included Acts and laws, regulations, policy documents, strategic plans, case studies, government reports and editorials. South Africa has a plethora of policies and regulations targeting major risk factors for diabetes and hypertension implemented in line with WHO ‘Best buys’ since 1990. A total of 28 policies, legislations, strategic plans, and regulations were identified - 8 on tobacco use; 7 on harmful consumption of alcohol; 8 on unhealthy diet and 5 on physical inactivity - as well as 12 case studies, government reports and editorials. There is good progress in policy formulation in line with the ‘Best buys’. However, there are some gaps in the implementation of these policies and programs.

**Conclusion:**

Curbing the rising burden of NCDs requires comprehensive strategies which include population-level interventions targeting risk factors for diabetes and hypertension and effective implementation with robust evaluation to identify successes and ways to overcome challenges.

**Supplementary Information:**

The online version contains supplementary material available at 10.1186/s12889-021-11910-6.

## Background

According to the recent report from the World Health Organisation (WHO), non-communicable diseases (NCDs) are increasing globally and are the leading cause of premature deaths with an estimation of 41 million people dying from cardiovascular diseases, cancer, diabetes among other NCDs globally [1]. Low- and middle-income countries (LMICs) bear a high burden of NCDs with almost 70% of NCD related deaths [2]. The South African Demographic Health Survey (SADHS) cited in the national NCD Strategic plan (2020–2025) found that both women and men with diabetes are obese (24% of women and 23% of men) [3]. In 2012, the prevalence of hypertension in South Africa was estimated at 35% among people aged 15 years and above [4].

Cardiovascular disease (CVD) related risks such as hypertension is the key driver for morbidity and mortality world-wide, affecting approximately 1 billion people [5]. In Sub-Saharan Africa (SSA), it is estimated that 10 to 20 million people may have hypertension out of 650 million people [5]. Furthermore, a study conducted in four SSA countries including South Africa (SA) shows that the prevalence of hypertension was high (25.9%) with almost half of the population unaware of having hypertension [6]. The economic burden of treating NCDs such as hypertension is quite high with an estimated cost of over 8 billion South African Rands annually [1]. The accumulated economic losses to SA’s “gross domestic product between 2006 to 2015 due todiabetes, stroke and coronary heart disease were estimated at R26 billion” [3]. A study conducted by the World Economic Forum and the Harvard School of Public Health cited in the WHO report [7] shows that LMICs account for 83% of the NCDs (as measured by disability-adjusted life years). The burden of deaths and prolonged disability related to NCDs has a considerable economic impact on households, industries, and societies through healthcare related cost and via losses in income, productivity, and human capital [7]. To curtail such human and economic losses, specific interventions must be implemented to effectively tackle NCDs and their underlying risk factors.

There is a growing body of evidence that highlights both health and economic gains of interventions at population-level. These population-level interventions include interventions that promote low consumption of tobacco, alcohol, and salt; improved awareness of healthy lifestyle; increased excise taxes; and enhanced regulation [8]. The WHO has developed cost-effective interventions called ‘Best buys’ that can be delivered at primary healthcare level. These interventions focus on promoting health and preventing disease by targeting risk factors of NCDs through increasing tobacco taxes; restricting alcohol advertising; reduction of salt, sugar, and fat in food products; access to vaccine for cervical cancer for young girls; treating hypertension and diabetes among others [8]. The WHO argues that these interventions are likely to save “10 million lives by 2025 and prevent 17 million strokes and heart attacks by 2030” [1].

Population-level interventions refer to policies or programs that aim at mitigating the distribution of health risk by addressing the underlying socio-economic, environmental, behavioral or cultural conditions in which people live and work [8]. Some interventions delivered in settings such as schools, and workplaces, can also be considered as population-level interventions. To accelerate national efforts to address NCDs, the World Health Assembly adopted a comprehensive global monitoring framework with 25 indicators and nine voluntary global targets for 2025 [2]. All WHO member states, of which SA is part of, through their ministries of health developed national NCD targets which informed the development and implementation of policies and interventions to be achieved through a 5-year SA national strategic plan (2012–2017). The latter is under review by various stakeholders in order to inform another 5 year strategic plan (2020–2025) [3]. Hence, it is imperative to take stock of the status of population-level interventions targeting risk factors of diabetes and hypertension, and NCDs at large at country level in order to inform the planned new NCDs strategic plan (2020–2025).

This document review forms part of a larger study on a “Situational analysis on population-level interventions targeting risk factors for diabetes and hypertension in SA”. The purpose of this document review was to identify all population-level interventions targeting risk factors for diabetes and hypertension implemented in SA. It focuses on population-level interventions at the level of governmental or political jurisdictions only, e.g., cities, regions, countries. We also looked at whether current population-level interventions targeting NCDs with specific emphasis on diabetes and hypertension are in line with the recommended WHO ‘Best buys’, and their implementation process (i.e. feasibility, acceptability, process and impact evaluation).

## Methods

This study used a document review methodology, which is one of the research methods commonly used in the field of Health Policy Analysis (HPA) [9]. The Centre for Diseases and Control defines document review as a research method whereby data is gathered from existing documents [10]. Kayesa and Shung-King in their systematic review argue that document review is a systematic way of reviewing existing documents (accessible online or grey literature) in order to understand and measure policy actions against what was stated or planned for [11]. Although there are other research methods employed in HPA, document review is the most convenient method used in LMICs [9].

A review of relevant reports, journal articles, or policy documents was conducted at the end of 2019. Documentation from government reports that contained information on the planning, implementation and evaluation of population-level interventions targeting diabetes and hypertension, and NCDs in general were considered. The WHO ‘Best buys’ [8] was used as framework to conduct the document review. These population-based interventions were grouped into three categories namely supportive policies, programs and enabling environments. The WHO Global Strategy for Diet, Physical Activity, and Health (DPAS) [12] defines these interventions as follow:

- *Supportive Policies* refer to fiscal, legislative and regulatory measures that can target risk factors for diabetes and hypertension. These policies could be trade and agricultural policies that promote healthy diets.
- *Supportive Programs* refer to national, district or community-based programs that reach people where they live, study, work, and play. These programs could be government or non-governmental health facilities offering diet and physical activity counselling for obesity or diabetes.
- *Supportive Environment / enabling environments* refer to activities tailored to influence the creation of environments in which healthy choices are the easier option for people. These could include cycling lanes, public gyms, etc. to promote physical activities.

Databases used to run the search included Pubmed, Ebscohost, Google scholar, Scopus, and the Cochrane database. Search terms included diabetes/hypertension/physical activity/nutrition/alcohol consumption/tobacco smoking/programs/interventions/South Africa (Additional file 1). There was no restriction of date and language applied to the search. An excel spreadsheet was designed to extract relevant information relating to what interventions have been implemented, their coverage, target audience and if they have been any process or impact evaluation of the identified interventions. Data extraction was done in duplicate (JN and LH).

The identified population-level interventions were grouped in line with WHO ‘Best buys’, and content analysis was conducted according to the major risk factors of NCDs i.e., tobacco use, harmful consumption of alcohol, unhealthy diet/nutrition and physical inactivity’. Obesity was considered as risk factor for both diabetes and hypertension, and identified population-level interventions targeting obesity were related to unhealthy diet and physical inactivity. Progress of the implementation of WHO ‘Best buys’ was done by checking whether the formulated supportive policies are in line with WHO ‘Best buys’ (content wise).

## Results

### Description of reviewed documents

**Fig. 1:**
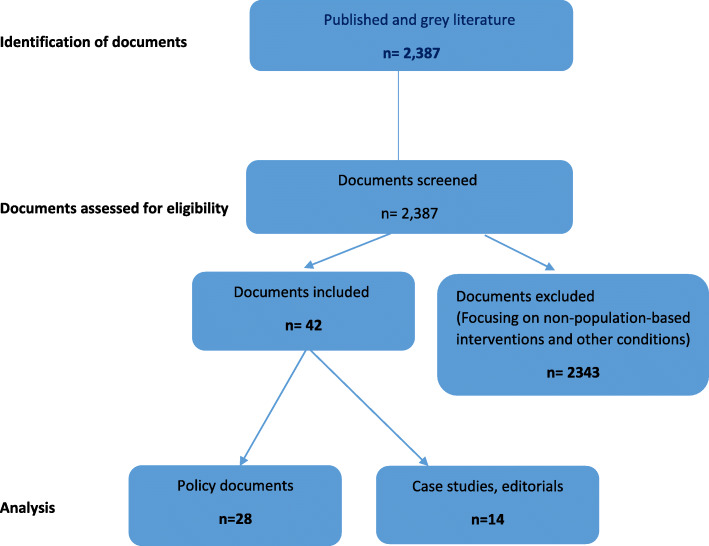
Flow diagram illustrating the document selection process

A total of 2387 records were retrieved (published and grey literature) and 42 documents were selected and reviewed (Fig. 1). Documents reviewed included 28 policies, legislations, strategic plans, and regulations (Table 1) and 14 case studies, government reports and editorials (Table 2). These were grouped into two categories namely Supportive Policy interventions; and Supportive Programs and Enabling environments given that the interventions related to ‘Enabling environments’ were few and linked to programs.

**Table 1:** Summary of Policy interventions targeting risk factors for diabetes and hypertension in South Africa

Risk factors	Policies	Year of inception																													
		1989	1990	1991	1992	1993	1994	1995	1996	1997	1998	1999	2000	2001	2002	2003	2004	2005	2006	2007	2008	2009	2010	2011	2012	2013	2014	2015	2016	2017	2018
*Tobacco use*	Tobacco Products Control Act 21					X																									
	Tobacco Products Control Regulations						X																								
	Tobacco Products Control Amendment Act 23											X																			
	Tobacco Products Control Amendment												X																		
	Tobacco Products Control Amendment Act 25																			X											
	Tobacco Products Control Amendment Act 28																				X										
	Tobacco Products Control Amendment regulations																							X							
	Control of Tobacco products and electronic delivery systems Bill																														X
*Harmful use of Alcohol*	Liquor Products Act 60	X																													
	National Liquor Act 59															X															
	National Liquor Regulations																X														
	Western Cape Liquor Act																				X										
	National Drug Masterplan (2013–2017)																									X					
	Gauteng Liquor Act																									X					
	City of Cape Town Liquor bylaw																										X				
	The National Road Traffic Act 93 Section 65								X																						
*Unhealthy diet*	Integrated Food security strategy														X																
	Regulations relating to trans-fat in foodstuffs																						X								
	Roadmap for Nutrition in South Africa 2012-16																								X						
	Salt Reduction Regulations																									X					
	Nutrition and Food Security Policy																									X					
	Strategy for Prevention and Control of Obesity (2015-2020)																											X			
	Taxation of Sugar Sweetened beverages Bill		2																										X		
*Physical inactivity*	Schools Act 84								X																						
	National Sports and Recreation Act										X																				
	Promotion of Physical Activity in Older persons																							X							
	National Sports and Recreation strategic plan (2012-2016)																								X						
	National Strategic Plan for NCDs (2013-2017)																									X

**Table 2:** Summary of case studies, government documents and editorials (*n* = 12)

Title	Type of paper	Citation
How will South Africa’s mandatory salt reduction policy affect its salt iodisation program? A cross-sectional analysis from the WHO-SAGE Wave 2 Salt and Tobacco study	Case study – cross sectional study	[13]
The evolution of non-communicable diseases policies in post-apartheid South Africa	Case study – Policy review through documents review supplemented by qualitative data	[14]
A hidden menace: Cardiovascular disease in South Africa and the costs of an inadequate policy response	Case study – Policy review undertaking document review	[15]
Analysis of non-communicable diseases prevention policies in Africa (ANPPA) – A case study of South Africa. A technical research report developed for the African Population & Health Research Centre (APHRC)	Case study – Technical report	[16]
South African Health Review - Diet-related non-communicable diseases in South Africa: determinants and policy responses	Case study – Policy review undertaking document review	[17]
Eating Better for Less: A National Discount Program for Healthy Food Purchases in South Africa	Case study- cross- sectional study	[18]
National School Nutrition Program South Africa.	Government Report	[19]
Department of Agriculture of South Africa 2012: Integrated food security strategy for South Africa	Government Report	[20]
South Africa’s salt reduction strategy: Are we on track, and what lies ahead?	Editorial	[21]
Evaluation of a Mass-Media Campaign to Increase the Awareness of the Need to Reduce Discretionary Salt Use in the South African Population	Case study – Cross sectional survey	[22]
Sugar-based beverage taxes and beverage prices: Evidence from South Africa’s Health Promotion Levy	Case study – Process evaluation	[23]
The distributional impact of taxing sugar-sweetened beverages: Findings from an extended cost-effectiveness analysis in South Africa	Case study - Economic evaluation	[23]
The effect of excise tax increases on cigarette prices in South Africa	Case study - Economic evaluation	[24]
Support for alcohol policies from drinkers in the City of Tshwane, South Africa: Data from the International Alcohol Control study	Case study - Cross sectional survey	[25]
Western Cape on Wellness (WOW!) Healthy Lifestyles Initiative Design and Outcome Evaluation of Phase 1 Pilot Implementation	Case study – Evaluation study	[12]

There is a plethora of available policies and regulations targeting major risk factors for diabetes and hypertension that have been in existence since 1990. These major risk factors include unhealthy diet, tobacco use, harmful alcohol use and physical inactivity; and were based on the WHO ‘Best buys’ [8].

There were 8 policies on tobacco use (smoking); 7 policies on harmful consumption of alcohol; 8 policies on unhealthy diet which include the National strategic plan for prevention and control of obesity and 5 policies on physical inactivity (Table 1). Table 3 provides a summary of the key strategies included in these policies and regulations.

**Table 3:** Key strategies included in the policies and regulations

Risk factor	Strategies
Tobacco use	Annual tax increases Advertisements of tobacco products banned. Tobacco smoking in public buildings banned. Allocation of smoke-free zone Health information and warnings
Unhealthy diet	Tax sugar-sweetened beverages Reduced salt intake The trans-fat content of any oils and fats cannot exceed two grams per 100 g, according to South African legislation. Products with higher trans-fats levels are prohibited from entering or being sold in the country
Harmful alcohol use	Zero tolerance with regards to “drink and driving”. Taxation Normalisation of the previously illegal drinking houses (Sheebens) Regulating drinking hours Change of the legal age for drinking from 18 years to 21 years. Banning alcohol advertising
Physical inactivity	Public awareness of physical activity Cycling lanes on major public roads

Supportive programs considered in this review are current programs targeting risk factors for diabetes and hypertension in line with the WHO ‘Best buys’, at governmental jurisdictions level (i.e., Cities, Provincial or National level). South Africa has several comprehensive programs that target a the four major risk factors and most of these programs focus on unhealthy diet, tobacco use and physical inactivity.

However, many of these programs have not been implemented across SA and evaluated to determine the impact on the occurrence and control of diabetes and hypertension, and NCDs prevention at large. Overall, 13 supportive programs were identified as currently implemented in SA, of which 6 focus on unhealthy diet [16, 18–20, 22, 26]; 3 on tobacco smoking [27–29] and 4 on physical inactivity [24, 30–32]. Table 4 summarises the programs, the risk factors, description, and findings from studies. Table 5 illustrate the progress of implementation of WHO ‘Best buys’ in South Africa to date.

**Table 4:** Summary of supportive programs for addressing unhealthy diet, smoking and physical inactivity

Risk factors	Programs	Description	Coverage	Target population	Key findings
***Tobacco use***	National Quit Line / National Council Against Smoking	A telephonic advice service on how to quit smoking is provided during office hours. They can also post a personal guide to quitting.	National	Smokers	Not evaluated
	CANSA’s eKick Butt program	Online smoking cessation program through which a series of emails, surveys and downloads guide and mentor a person. This program supplies a series of handy tools to help to quit for good.	National	Smokers	Not evaluated
	Smokenders	Over a period of six weeks participants attend six weekly group meetings lasting 2 h each. It consists of One-on-one counselling.	Cape Town, Pretoria and Johannesburg	Smokers	Not evaluated
***Physical inactivity***	Move for Health Day	Annual campaign conducted on the 10th of May led by the Department of Sports and Recreation in collaboration with the DOH. Encouraging cost effective physical activities such as walking, regular exercise and other extramural activities.	National	Young and old	No evaluation conducted
	National recreational day	An annual campaign conducted the 1st Friday of October since 2014. The campaign targets all Citizens to be physically active.	National	Young and old	No evaluation conducted
	Big walk Day	Annual based initiative led by the Department of Sports and Recreation. The purpose of the day is to promote participation in sport and recreation.	National	Young and old	Started 2012, implemented in all provincial capital cities since 2015. Grown from less than 2000 participants to 30,000 in 2017
	Western Cape on Wellness (WOW!)	A health promotion program designed by the Western Cape Provincial Government Department of Health to address modifiable risk factors associated with NCDs. The WoW! Initiative incorporates a novel transversal partner approach, involving “health champions”, to help group members activate healthy lifestyles, in workplace, school and community settings.	Western Cape Province	Multi-sectoral (WCP, Universities and NGOs)	Over 80% presenting at baseline were either overweight or obese, over 50% from schools and community groups were hypertensive, and less than 1 in 4 were meeting international recommendations for physical activity (> = 150 min/week). Improvements were seen in systolic and diastolic blood pressure at 3 months, and systolic blood pressure at 6 months; the proportion of members presenting with hypertension at 3 months was halved. Improvements in self-reported healthy eating, physical activity, quality of life, and general health status, and reductions in waist circumference at 6 months, and in time spent sitting. Self-reported smoking changed from 10% at baseline to 0% at 6 months.
***Unhealthy diet / nutrition***	National School Nutrition Program	The program provides one nutritious meal to all learners in poorer primary and secondary schools. It also teaches learners and parents on ways of living a healthy lifestyle and promoting development of school vegetable gardens.	National	Schools learners and teachers	The program provides meals to > 9 million children in public schools.
	Integrated nutrition program	Multi-sectorial program, which includes the Departments of Health, Social Development and Agriculture.	National	Children	No evaluation conducted
	Healthy Food -Discovery Health	Benefit to members of Discovery (private health insurance company) whereby members receive up to 25% cash back on healthy food purchases.	National	Discovery members	Participation associated with more consumption of healthy food (fruits/vegetables and wholegrain foods) and less consumption of unhealthy food (high sugar/salt foods, fried foods, processed meats, and fast-food).
	Making the Difference through Nutrition	The program combines outcomes-based education, interactive classroom activities and informative parent workshops, which positively influence the lifestyles and well-being of young learners and their communities. One of the primary focuses of this initiative is to teach learners not only the importance of regular physical activity, but also how to become more physically active in a fun, creative and sustainable manner.	Some provinces	Woolworths	Woolworths and SSISA have produced a DVD to educate children, caregivers, parents and teachers about how to make physical activity an integral part of the lives of children. In the DVD the presenters give valuable insights into the extensive benefits of physical activity, as well as practical advice on how to improve children’s physical activity levels at home and at school. The 1 km Health Track and the dynamic group dance (Dance for Fun), provide two inspiring examples of how exercise can be accessible and fun
	Salt watch Campaign	Salt Watch is a mass-media campaign to increase public awareness related to the association between a high salt intake, blood pressure and cardiovascular disease, and to highlight the need to reduce discretionary salt intake. Television and radio advertisements aimed at strengthening the advertisement message and providing additional information and education materials regarding salt reduction.	Some provinces (KZN, Gauteng & EC)	Heart and Stroke foundation	550 women participated in the baseline study and 477 in the follow-up survey. Knowledge, attitudes and behavior change occurred with a significant move towards considering and initiating reduced salt consumption. Post intervention, more participants reported that they were taking steps to control salt intake (38% increased to 59.5%, *p* < 0.0001). In particular, adding salt while cooking and at the table occurred significantly less frequently.

**Table 5:**
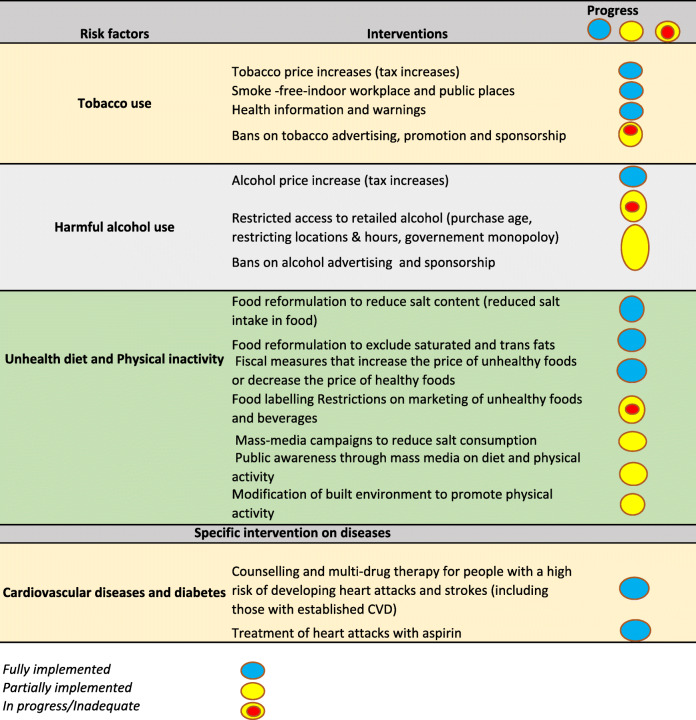
Implementation of “WHO Best Buy’ Interventions in South Africa to date

### Implementation and impact of population-level interventions in South Africa

Ndinda and Hongoro’ report [16] on the analysis of NCDs prevention policies in SA highlights that the implementation of initial regulations on tobacco products contributed to reduction in tobacco smoking by 26% in the period between 1993 and 2000. This reduction in smoking was mainly seen among young people aged 16–24 indicating a decline from 24 to 19% in the same period [24]. However, the rate of smoking has been stagnant since 2010. However, there is no available data that explain the reasons for stagnation in smoking among South Africans.

The annual tax increase on tobacco products has the potential to discourage people who might want to adopt smoking but also to incentivise those who have been trying to quit [16]. According to Linegar and Walbeek [24], in 2018 the excise tax on tobacco products in SA was at 52% below the recommended WHO Framework Convention on Tobacco Control (FCTC). Hence, the NDoH has revised its regulations to enforce plain packaging and clean air regulations, regulate e-cigarettes, and increase taxes to revitalise efforts to reduce tobacco use [16].

In 2010, the WHO estimated an average annual per capita consumption of alcohol for SA (persons over 15 years of age) as 11 l; and the numbers of heavy episodic drinkers as 26%. The policies on harmful alcohol use focus more on the regulation of alcohol production and distribution which address mainly trade and industry concerns than prevention of NCDs [16]. They reported that re-enforcement of these policies is lacking and thus the difficulty to measure the impact of such policies/regulations on reduction of alcohol consumption.

Unhealthy food environments, including limited access to and affordable healthy food contribute to consumption of these unhealthy food. Healthier food options are relatively costly ranging from 10 and 60% more compared to the prices of unhealthy foods at retail outlets [3]. Drawing on best practice from high income countries in preventing and controlling NCDs by targeting unhealthy diets, the South African government introduced taxation on sugar-sweetened beverages (SSBs) from 1st April 2017 [16]. Beverages such as soft drinks, fruit juices, energy drinks and vitamin water were levied [17].

Stacey et al. [33] in their study highlights that no price increase among non-taxed beverages and that there was a significant price increase for carbonated drinks. The latter was identified as the largest taxed product category of SSBS. An economic evaluation by Saxena et al. [23] found that “10% SSB tax increase would avert an estimated 8000 Type 2 Diabetes Mellitus (T2DM)-related premature deaths over 20 years, with most deaths averted among the third- and fourth-income quintiles”. The same study estimated that “32 000 of T2DM related cases of catastrophic expenditures and 12 000 cases of poverty would be averted” [23]. However, there are no accessible data at population-level that could determine the impact of SSB taxation on the occurrence of diabetes.

In 2011, the former Minister of Health Dr. Mostoledi pioneered the legislation for salt reduction in food products and encouraging South Africans to be conscious of their salt intake. Studies show that reformulation of salt in food products such as bread has the potential of saving 6500 lives annually [16]. By 2013, the salt reduction regulations were adopted as an intervention targeting hypertension [14]. The NDoH granted a 3-year period to food industries to tease out ways of reformulating and producing lower salt products acceptable to consumers. It has been estimated that SA’s salt reduction policy will reduce 11% of deaths from heart disease per year and save the government approximately ZAR 713 million per year in healthcare costs [21]. At the individual level, healthcare cost savings could prevent 2000 households being pushed into poverty [21].

In addition to legislation, Webster et al. [21] highlights some priority areas for continued action on salt reduction – active monitoring of formal and informal food industry, improved awareness on discretionary salt intake and addressing behaviour change and other social determinants of health.

Furthermore, a study conducted by the WHO-SAGE Wave 2 Salt & Tobacco highlights the need to closely monitor the iodine status of populations as the measures of salt reduction are implemented [13].

The Bill on Trans-fatty acid [34] focused on reducing trans-fats content in certain processed and prepared foods currently for sale in SA, because trans-fatty acids significantly increase the risk of CVD. The trans-fat content of any oils and fats cannot exceed 2 grams per 100 g, according to South African legislation [34]. Products with higher trans-fats levels are prohibited from entering or being sold in the country. The legislation covers any oils and fats “either alone or as processed foods, which are intended for human consumption or assumed to be intended for human consumption, in the retail trade, catering businesses, restaurants and institutions” [34]. Manufacturers and retailers were given 6 months to comply and reduce the trans-fat content of their products to the required 2 g per 100″ [16].

Currently, the guidelines on Advertising and Labelling of Foodstuffs (under revision), which were promulgated under the Foodstuffs, Cosmetics and Disinfectants Act, 1972 (Act 54 of 1972) do not address the labelling of trans fats, especially industrially produced trans fatty acids [15]. The NDoH has been monitoring the labels of food products and the food industry has complied to some extent. However, there is no systematic assessment on how well industries have complied with food labelling and whether food libelling has influenced behaviour change or consumer patterns of certain food [14]. Therefore, empirical studies are needed to determine the impact of food labelling interventions on consumers’ behaviour.

Other relevant national level-policies that shape provincial and community-level actions impacting food environments are the Integrated Food Security Strategy, the Integrated Nutrition Programme, the National School Nutrition Programme, and the National Policy on Food and Nutrition Security. However, collectively these policies frame food insecurity as they do not take account of environmental issues or spatial contexts around access and affordability to nutritious food [17]. The SA sports policy was formulated in 1998 with a focus on transformation of sport and racial representation in competitive sport. The National Sport and Recreation Strategic Plan 2012 [35] lists appropriate objectives to tackling physical inactivity in the general population, aiming for a 10% increase in the uptake of physical activity at population-level by 2020. However, there is a lack of interventions geared to an enabling environment countrywide as well as monitoring and evaluation (M&E) systems to measure the impact of these interventions [16].

### Challenges for the implementationof population-level interventions in South Africa

Limited funding allocated to provinces and NGOs was identified as the main hindrance to effective implementation of population-level interventions as well as lack of strong monitoring and evaluation systems at grassroots level in order to determine the impact of these interventions [17]. Most of supportive programs and enabling environment activities are mainly implemented at provincial level by NGOs while government structures focus on policy formulation.

Also, there is a conflict of interest between the national trade and investment policy vis-a-vis the national health policy whereby trade and foreign direct investment have a tendency of promoting the influx of large amounts of processed foods and sugary beverages, giving a certain level of power to fast food companies like McDonalds and Burger King [17].

Finally, lack of inter-sectoral approaches in implementing these population-level interventions targeting risk factors of diabetes and hypertension has been identified as another impediment to effective policy implementation (16; 29).

## Discussion

Both hypertension and diabetes, are major contributors to morbidity and mortality worldwide [36]. In 2013, the SA government committed to reduce by at least 25% the relative premature mortality (under 60 years of age) from NCDs by 2020 through its strategic plan for NCDs [37]. The findings of this document review show that the SA government has made progress with policy formulation and implementation of population-level interventions targeting diabetes and hypertension, and NCDs prevention at large as recommended by WHO ‘Best buys’. The findings of this review echoes the findings from the WHO report on the NCDs progress monitor [1].

Supportive programs identified in this document review focus on unhealthy diet, tobacco use and physical inactivity. The coverage of these supportive programs ranged from national, provincial to city level. There are fewer supportive programs targeting harmful consumption of alcohol. This could be due to the potential economic conflicts between health gains vs economic gains which might result in opposition from industries as it has been reported in other African countries [38, 39].

The findings of this document review also highlight that despite interventions addressing tobacco consumption such as taxation, a ban on smoking in public spaces and cigarette advertising, and written warnings on cigarette packaging, there is a gap in policy legislation when it comes to smoking in work areas where non-public servants such as domestic workers, gardeners and others operate [17]. Similarly to other SSA countries [38], SA is still lagging behind in achieving the implementation of the full WHO FCTC. This could be due to the inherent conflicts of priorities between government departments such as NDoH and department of Trade and Industry on regulations related the economic gains [39, 40]. Although SA is one of the few countries in LMICs with many supportive policies and programs on NCDs including diabetes and hypertension in line of WHO ‘Best buys’, the extent of implementation and their impact on the overall NCDs related outcomes is a big question that still needs to be answered.

Lack of multi-sectoral approaches in LMICs is a contributing factor to non-effective implementation of supportive interventions [39]. This has been echoed by various researchers in LMICs [39, 41, 42]. Hence, there is a need for multi-sectoral and bottom-up approaches for effective implementation of population-level interventions on NCDs. Achieving global and national targets for physical activity, requires a multi-sectoral collaboration between transport, urban planning, recreation, and sports and education departments as well as the South African policy services (SAPS) to create safe environments that are conducive to physical activity for all age groups [43]. A study conducted in Ghana and Cameroon [44] shows that there have been a number of programs striving to create enabling environments to promote physical activities such as creation of fitness clubs, community works led by church groups but these were mainly covering a particular class of the society – urban areas and middle income groups.

Supportive environment interventions targeting risk factors for diabetes and hypertension implemented at country level seems not to be well documented. This could be due to the fact that most of supportive environment activities are implemented at community and individual level with little or non-existence of systematic documentation of such programs [45]. In addition, documentation on the process of implementation and evaluation (M&E) is missing in the literature. This has been observed in other LMICs such as Kenya, Togo, Cameron, Malawi and Iran where the implementation of WHO ‘Best buys’ have been largely focusing on policy and programs than creating enabling environments that target risk factors related to diabetes and hypertension [39, 41]. Lack of M&E plans for population-level interventions and for NCDs in general have been reported in other SSA countries such as Zambia, Kenya, Malawi and Cameroon [38, 39].

The findings of our document review show that limited funding for population-level interventions and NCDs prevention policies and programs has been one of the contributing factors to poor implementation of these policies and programs. Bourdeaux and colleagues argue that limited funds for NCDs prevention and control is due to the fact most LMICs countries get their funding from NGOs [39]. Consequently, limited funding for the implementation of NCDs strategic plans has a direct impact on establishment of M&E systems to assess the effectiveness and impact of the policies and programs emanating from these NCDs strategic plans.

### Study strengths and limitations

Document reviews are commonly used in the field of HPA [9, 11], particularly in LMICs, as is the most convenient approach to understand the measure what policy actions against what was stated and planned. Key stakeholders involved with development and formulation of NCDs policies at national and provincial levels, helped us in accessing some of the policy documents that were not in the public domain and assisted in inclusion of document to review [11]. We used a comprehensive approach to identify relevant documents and to extract data in a standardised manner. The data extraction of included studies was based on the WHO ‘Best buys’ which provided a focus lens for taking stock of population-level interventions that have been proved to be effective. However, not all documents were in the public domain and accessible. The findings of this study are limited to what has been documented. It’s important to note that the findings of this document review could be enhanced through input from key role players on policy implementation process, related successes and challenges. Also, there is a scarcity of population-level data and empirical studies that assess the effectiveness and the impact of supportive policies, programs and enabling environment targeting diabetes and hypertension on the prevalence and occurrence of diabetes and hypertension in South Africa. Hence, there is a need for future studies to determine the impact of WHO ‘Best buys’ interventions on prevention and control of NCDs in general.

## Conclusion

South Africa has a growing burden of NCDs particularly diabetes and hypertension. Mortality and prolonged disability associated with NCDs have a considerable economic impact on households, industries and societies, both via the consumption of health services and via losses in income, productivity and capital formation. South Africa has done relatively well in including WHO ‘Best buys’ interventions in policies and with implementation. As the National Department of Health is revising the NCDs strategic plan (2020–2025), a critical engagement between inter-government departments and private sector as well as the public in formulating and implementing supportive policies, programs and enabling environments is paramount. Empirical studies are needed to determine the impact of population-level interventions on prevention and control of hypertension and diabetes, and other NCDs.

## Supplementary Information

**Additional file 1.** Pubmed search strategy.

## Data Availability

All data relevant to the study are included in the article or uploaded as supplementary information.
