# Supplementary figures and images for: Population-level interventions targeting risk factors of diabetes and hypertension in South Africa: a document review
Source: BMC Public Health. 2021 Dec 14;21:2283. doi: 10.1186/s12889-021-11910-6 (PMC8670282; doi:10.1186/s12889-021-11910-6)

# Additional file – Pubmed search strategy

PUBMED- MEDLINE - 27 Nov 2019


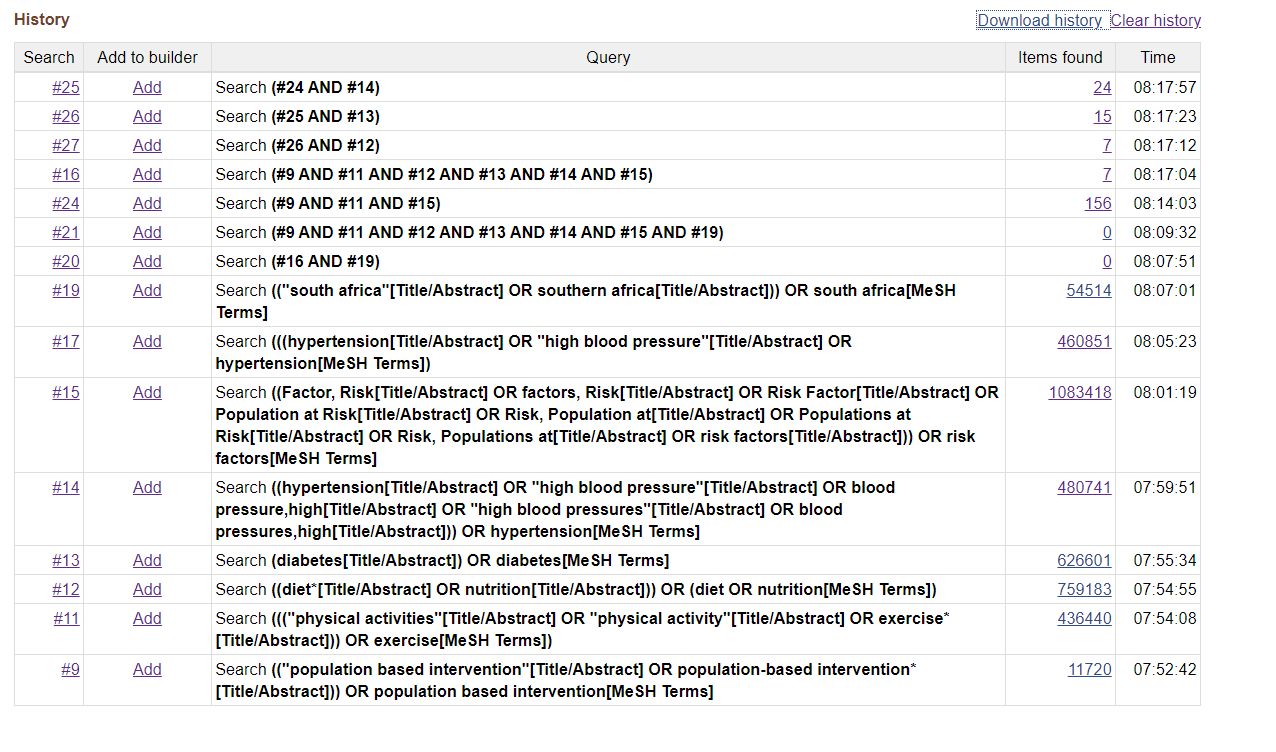

Supplement: Supplementary file 1 — Additional file 1. Pubmed search strategy. [file 12889_2021_11910_MOESM1_ESM.docx]
